# Supplementary material for: Development of Reverse Transcription Recombinase Polymerase Amplification (RT-RPA): A Methodology for Quick Diagnosis of Potato Leafroll Viral Disease in Potato
Source: Int J Mol Sci. 2023 Jan 28;24(3):2511. doi: 10.3390/ijms24032511 (PMC9916786; doi:10.3390/ijms24032511)
Supplement: Supplementary file 1 [file ijms-24-02511-s001.zip › ijms-2161052-supplementary.pdf]

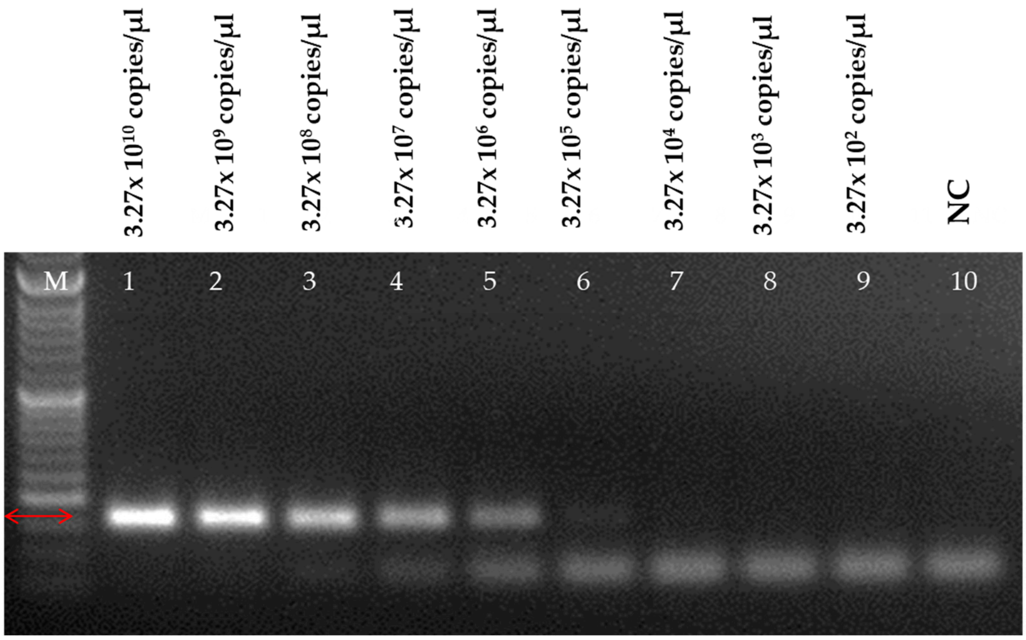

Supplementary Figure S1. Limit of detection of PLRV using ten-fold serial dilution starting from 3.27x 10^{10} copies/µl. Lane 1-9, plasmid dilution ranging from 3.27 × 10^{10} to 3.27 × 10^{2} copies/µl. Lane 10, NC (water control). M, 50bp DNA ladder. The limit of copy numbers using serially diluted plasmid was also determined which came to 3.27 × 10^{6} copies/µL

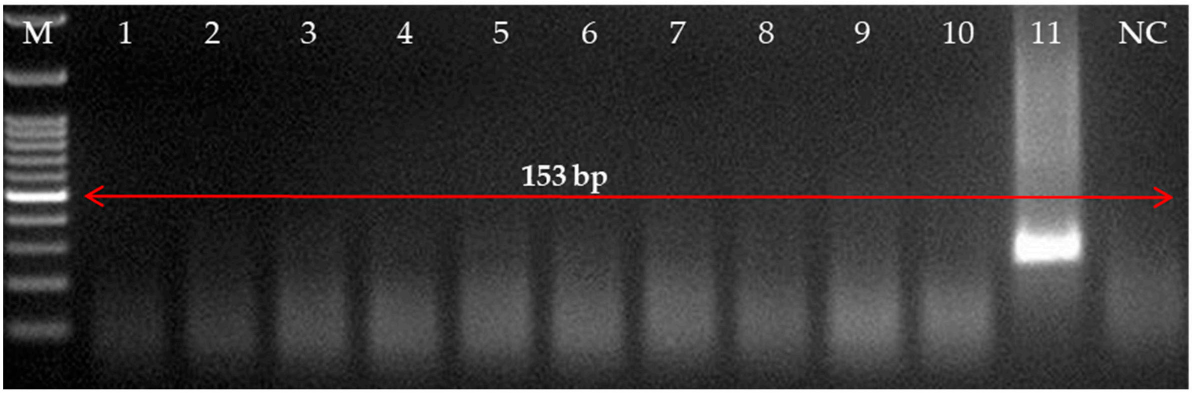

Supplemental Figure S2. Specificity analysis of one step RT-RPA reaction for the detection of PLRV with primer pair C (LRRPAF3/R3). DNA for ToLCNDV (1) infected plants and RNA extracted from PVS (2), PVA (3), PVM (4), GBNV (5), PSTVd (6), PVX (7), PVY^{O} (8), PVY^{NTN} (9), PVY^{N} (10), PLRV (11) infected plants along with NC-water control were used. M-50bp ladder. There was no match observed with other viruses such as potato virus A (PVA), potato virus M (PVM), PVS, tomato Leafcurl New Delhi virus (ToLCNDV), PVX, PVYNTN, PVYNT, PVYO, groundnut bud necrosis virus (GBNV) and potato spindle tuber viroid (PSTVd) during the specificity analysis experiment
